# 

**Published:** 1973-01

**Authors:** 


Bristol Medico-Chirurgical Journal Vol. 88 (i) No. 32[illegible]

### Contents

The Perfectionist ... ... ... ... 1

C. C. Morgans

Adult Acute Leukaemia — a Suitable case for treatment? ... ... ... 11

T. J. Hamblin and J. Verrier Jones

Viscum Album — A possible treatment of Cancer? ... ... ... ... ... 17

M. R. Evans and A. W. Preece

The Medical Library ... ... ... ... 21

Printed by F. Bailey & Son Ltd., Dursley, Glos.

